# Training trichiasis surgeons: ensuring quality

**Published:** 2014

**Authors:** Emily W Gower, Amir Bedri Kello, KH Martin Kollmann

**Affiliations:** Associate Professor: Wake Forest School of Medicine, Winston-Salem, USA. egower@wakehealth.edu; Senior Adviser on Eye Health: Light for the World, Addis Ababa, Ethiopia. a.bedri@light-for-the-world.org; Senior Advisor for NTDs: CBM, University of Nairobi, Nairobi, Kenya. khm.kollmann@gmail.com

**Figure:**
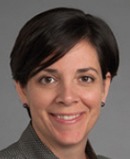
Emily W Gower

**Figure:**
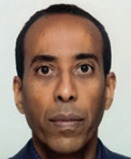
Amir Bedri Kello

**Figure:**
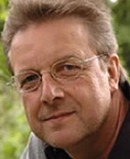
KH Martin Kollmann

The World Health Organization (WHO) has set 2020 as the target for the global elimination of blinding trachoma as a public health problem. To achieve this goal, more than 7 million trachomatous trichiasis (TT) operations are needed.^1^ Poor post-operative outcomes are a significant barrier, as they reduce the number of patients who are willing to come for treatment. High-quality, high-volume surgery is needed to reach the WHO target,^2^ and this begins with high-quality training.

The WHO endorses two procedures for the correction of trichiasis and the WHO manual *Trichiasis Surgery for Trachoma*^3^ (commonly referred to as the ‘yellow manual’) provides specific training guidelines for these procedures. This article is intended to provide general guidance for national programmes on training to encourage high-quality surgery.

Attention to the following core aspects will help to ensure high-quality outcomes.

## Trainer selection

Individuals selected to serve as TT surgery trainers should not only be highly-skilled, active TT surgeons themselves, but also should be good at training and supervising others. A good trainer should have:

- Patience
- Good communication skills
- An ability and willingness to provide constructive feedback.

Wherever possible, cascade training (in which individuals learn how to perform TT surgery and then teach their peers the procedure), should be avoided, as it is likely to jeopardise the quality of training.

## Trainee selection

All potential trainees should have a core set of prior experience, as described on page 49 of the yellow manual.^3^ They should also have a demonstrated ability to acquire new skills, a positive attitude and the potential to maintain high productivity and quality outcomes. Potential trainees should be carefully screened to ensure that they have good manual dexterity, excellent corrected near vision and depth perception, and are able to perform careful suturing.

## Programme duration

Training programmes typically range from 1–4 weeks. Rather than duration, the number of TT operations performed under supervision is more important.

## Classroom instruction

Classroom instruction should be based on the yellow manual and also should include teaching about the appearance of a good immediate post-operative outcome and how to correct any problems noted at that time.^4^ Trainees should learn that a poor immediate post-operative appearance can indicate potential unfavourable outcomes later on. Trainees should be aware of the most common unfavourable outcomes: under-correction (post-operative trichiasis), over-correction, pyogenic granuloma formation and eyelid contour abnormalities^5^, and they should be able to describe how to avoid and/or manage these outcomes.

## Skills practice prior to live surgery

All trainees should have the opportunity to practice critical steps of the procedure before performing surgery on live patients. Traditionally, practice opportunities were limited to oranges and gloves. Surgical mannequins, such as the HEAD START training system, available on the IAPB Standard List for TT surgery, now offer the opportunity to bridge the gap between classroom and live-surgery training. Trainees should not be allowed to conduct live surgery until they can adequately demonstrate the ability to:

- Hold and manipulate surgical instruments properly
- Make a straight incision of the appropriate depth and length
- Consistently take suture bites of the proper depth
- Place sutures parallel to each other
- Tie suture knots with proper tension
- Comprehend and implement instructions
- Critically self-reflect.

## Live surgery practice

Trainees should begin by watching the trainer perform at least two live TT operations. No more than two trainees should observe surgery at one time. Next, the trainee can begin performing individual aspects of the procedure under close supervision. The trainer should be prepared to step in when necessary, as the patient's safety and the surgical quality are of utmost importance. As the trainee becomes experienced, supervision can be gradually reduced, but the trainer should always evaluate the final outcome before the eyelid is patched.

**Figure:**
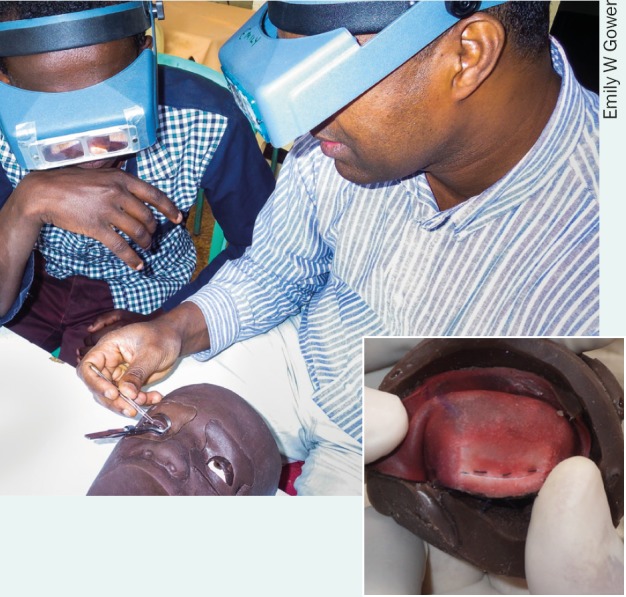
Use of a surgical mannequin (HEAD START) to train new trichiasis surgeons

## Certification

All those who complete the training process should undergo certification before being allowed to perform surgery independently. The yellow manual provides a certification checklist to guide independent, critical evaluation of trainees' skills, which is normally done during five independent operations performed under direct supervision of the trainer/examiner at the end of the training programme. During the process, the examiner should make an honest appraisal of the trainee's skills. No one should be certified unless they can successfully demonstrate all of the skills necessary to consistently provide high-quality surgery. In some settings, it may be appropriate if trainees who fail are given additional supervision and more practice followed by a repeat certification evaluation. However, if the trainee does not possess the appropriate skills to become a TT surgeon, she or he should be encouraged to find another career path.

After training, regular supportive supervision and monitoring are critical to any successful TT surgery programme. These aspects will be covered in a future article.

## References

[1] ICTC. 2020 INSight, the end in sight. International Coalition for Trachoma Control; 2011.

[2] ICTC. Global Scientific Meeting on Trachomatous Trichiasis. International Coalition for Trachoma Control; 2012.

[3] Merbs SL Resnikoff S, et al. Trichiasis Surgery for Trachoma. 2nd ed. Geneva: World Health Organization; 2013.

[4] Merbs SL Harding JC, et al. Relationship between immediate post-operative appearance and 6-week operative outcome in trichiasis surgery. PLoS Negl Trop Dis 2012; 6: e1718. 22802976 10.1371/journal.pntd.0001718 PMC3393656

[5] Gower EW West SK, et al. Definitions and standardization of a new grading scheme for eyelid contour abnormalities after trichiasis surgery. PLoS Negl Trop Dis 2012; 6: e1713. 22745845 10.1371/journal.pntd.0001713 PMC3383763

